# The potassium channel KCa3.1 represents a valid pharmacological target for microgliosis-induced neuronal impairment in a mouse model of Parkinson’s disease

**DOI:** 10.1186/s12974-019-1682-2

**Published:** 2019-12-26

**Authors:** Jia Lu, Fangfang Dou, Zhihua Yu

**Affiliations:** 1 Department of Pharmacology and Chemical Biology, Shanghai Jiao Tong University School of Medicine, 280 South Chongqing Road, Shanghai, 200025 China; 2 Basic Research Department, Shanghai Geriatric Institute of Chinese Medicine, Shanghai University of Traditional Chinese Medicine, Shanghai, 200031 China

**Keywords:** KCa3.1, Orai1, Microglia, Neuroinflammatory, Senicapoc

## Abstract

**Background:**

Recent studies described a critical role for microglia in Parkinson’s disease (PD), where these central nerve system resident immune cells participate in the neuroinflammatory microenvironment that contributes to dopaminergic neurons loss in the substantia nigra. Understanding the phenotype switch of microgliosis in PD could help to identify the molecular mechanism which could attenuate or delay the progressive decline in motor function. KCa3.1 has been reported to regulate the “pro-inflammatory” phenotype switch of microglia in neurodegenerative pathological conditions.

**Methods:**

We here investigated the effects of gene deletion or pharmacological blockade of KCa3.1 activity in wild-type or KCa3.1^−/−^ mice after treatment with 1-methyl-4-phenyl-1,2,3,6-tetrahydropyridine (MPTP), a mouse model of PD. MPTP-induced PD mouse model was subjected to the rotarod test to evaluate the locomotor ability. Glia activation and neuron loss were measured by immunostaining. Fluo-4 AM was used to measure cytosolic Ca^2+^ level in 1-methyl-4-phenylpyridinium (MPP^+^)-induced microgliosis in vitro.

**Results:**

We report that treatment of MPTP-induced PD mouse model with gene deletion or pharmacological blockade of KCa3.1 with senicapoc improves the locomotor ability and the tyrosine hydroxylase (TH)-positive neuron number and attenuates the microgliosis and neuroinflammation in the substantia nigra pars compacta (SNpc). KCa3.1 involves in store-operated Ca^2+^ entry-induced Ca^2+^ overload and endoplasmic reticulum stress via the protein kinase B (AKT) signaling pathway during microgliosis. Gene deletion or blockade of KCa3.1 restored AKT/mammalian target of rapamycin (mTOR) signaling both in vivo and in vitro*.*

**Conclusions:**

Taken together, these results demonstrate a key role for KCa3.1 in driving a pro-inflammatory microglia phenotype in PD.

## Background

As the second most common neurodegenerative disease, Parkinson’s disease (PD) is a multifactorial disorder that has a strong environmental component, which involves motor deficits including bradykinesia, impaired gait, muscle rigidity, and tremors [1]. PD is characterized neuropathologically by progressive dopaminergic neuronal loss in the substantia nigra pars compacta (SNpc), and dopamine modulators are used as the first-line therapeutic in PD. However, serious side effects were found during the dopamine modulator treatments [2].

Microglia, as the resident immune cells of the central nerve system (CNS), plays a key role in neurotoxicity by initiating the inflammatory response in PD neurodegeneration [3]. Upregulation of proinflammatory cytokines released by reactive microglia in the SNpc was demonstrated to be associated with PD [4]. Activation of neuroinflammatory microglia by interferon (IFN)-γ or lipopolysaccharide (LPS) induced reactive astrogliosis by secreting cytokines, interleukin 1 (IL-1), tumor necrosis factor (TNF), and complement component 1q [5], which can ultimately lead to neuronal loss during the process of neurodegenerative diseases [6]. Microgliosis was reported to be associated with the histological changes in PD brains and mouse models of the disease. 1-Methyl-4-phenyl-1,2,3,6-tetrahydropyridine (MPTP)-induced mouse model of PD has also shown SNpc microgliosis correlating with dopaminergic neurons loss [7]. Therefore, attenuation of the microglia-mediated neuroinflammation has become an important point to ameliorate the neurodegenerative process.

Plasma membrane ion channels are good candidates among the possible CNS neuroinflammatory modulators, which participate in regulating membrane potential and intracellular signaling in immune cells such as T cells, B cells, macrophages, and microglia [8, 9]. In this work, we investigated the role of the intermediate-conductance calcium-activated potassium channel KCa3.1, in shaping the microglia activation state in a mouse model of PD. In the CNS, KCa3.1 channels regulate glial cell migration and phagocytic activity in physiological and pathological conditions such as Alzheimer’s disease (AD), ischemic stroke, and spinal cord injury [10–13]. Microglial KCa3.1 potentiate the neuroinflammatory response induced by oligomeric amyloid-β and LPS treatment, while pharmacological blockade or gene deletion of KCa3.1 has beneficial effects in rodent models of ischemic stroke, AD, and multiple sclerosis, reducing inflammatory factors such as TNF-α and IFN-γ expression in the spinal cord or the brain tissue [14, 15]. The KCa3.1 inhibitor per se is indirectly neuroprotective via attenuating the gliosis.

However, the role of KCa3.1 in PD has not yet been determined. We hypothesized that KCa3.1 involved in microgliosis-induced SNpc neuronal loss and demonstrated that both genetic deletion and pharmacological blockade of KCa3.1 reduced dopaminergic (DA) neuron loss and improved the locomotor ability via reducing microgliosis-mediated neuroinflammatory cytokine production in PD. We report for the first time to our knowledge that KCa3.1 involves in store-operated Ca^2+^ entry (SOCE)-induced Ca^2+^ overload and endoplasmic reticulum (ER) stress via the protein kinase B (AKT) signaling pathway during microglia activation. Gene deletion or blockade of KCa3.1 restored AKT/mammalian target of rapamycin (mTOR) signaling both in vivo and in vitro*.*

## Material and methods

### Compounds and formulations

Senicapoc (ICA-17043; Target Molecule Corp., Boston, MA, USA) was dissolved in dimethyl sulfoxide (DMSO, Sigma-Aldrich, St. Louis, MO, USA) to provide a 100-mM solution, which was diluted in medium for in vitro studies. For oral administration to C57BL/6 mice, senicapoc was dissolved in a 10:90 (*v/v*) mixture of macrogolglycerol ricinoleate (Calbiochem®; Merck, Darmstadt, Germany) and water to provide a dosing solution for administration at 100 mg/kg. 1-Methyl-4-phenylpyridinium (MPP^+^) and MPTP were obtained from Sigma-Aldrich. All drug solutions were prepared on the day of the experiments.

### Animals

Animal experiments were conducted in accordance with NIH guidelines for the Care and Use of Laboratory Animals under a protocol that was approved by the Animal Experimentation Ethics Committee of Shanghai Jiao Tong University School of Medicine, Shanghai, China (ethics protocol number: A-2015-010). KCa3.1^−/−^ mice (B6;129S1-Kcnn4^*tm1Jemn*^/J) were purchased from the Jackson Laboratory. These mice were bred to C57BL/6 J mice for at least 10 generations. Twelve-week-old male or female C57BL/6J mice (SLAC Laboratory, Shanghai, China, 25–30 g at the start of the treatment) were housed in a temperature-controlled environment and maintained on a 12-h light/dark cycle (lights on at 6:00 am).

### MPTP mouse model

This mouse model for PD was induced by administration of MPTP (Sigma, St. Louis, MO) as described previously [16]. Twelve-week-old male or female C57BL/6J mice and KCa3.1^−/−^ mice were randomly divided into eight groups as described in Table 1.

**Table 1:** MPTP mouse model groups

Groups (*n* = 10–15/group)	Treatment (day1–5)
Control	Vehicle
MPTP	20 mg/kg, once daily, i.p. day1–5
MPTP+senicapoc	MPTP (20 mg/kg, once daily, i.p. day1–5) + senicapoc (100 mg/kg, once daily, p.o. day1–5, 1 h prior to MPTP exposure)
Senicapoc	100 mg/kg, once daily, p.o. day1–5
WT	Vehicle
KCa3.1^−/−^	Vehicle
WT+MPTP	20 mg/kg, once daily, i.p. day1–5
KCa3.1^−/−^+MPTP	20 mg/kg, once daily, i.p. day1–5

After repeated intraperitoneal injection of MPTP with or without oral senicapoc for 5 days, performance in the rotarod and open field tests was then evaluated daily for 7 days. On day 13, mice were sacrificed, and brains harvested for immunohistochemistry or western blotting.

### The rotarod test

#### The senicapoc treatment group

A modification of the procedure described by Rozas was employed using a rotarod apparatus (IITC Life Science, Woodland Hills, CA, USA) [17]. In general, mice were tested on rotating rods at 8, 12, 16, 20, 24, 28 rpm once daily for 7 days. Duration of each speed does not exceed 150 s. The resting period was 5 min between each speed for alleviating the stress and fatigue

#### The genetic KCa3.1 deletion treatment group

For the rotarod test, mice were trained before MPTP treatment at a speed of 20 rpm to obtain a stable baseline. Then, at a constant speed of 28 rpm, the time each mouse remained on the rod was recorded during a 5-min trial once daily for 7 days after completion of MPTP treatment. On each day, mean time on the rod over three individual trials was recorded, with mice allowed to recover in their home cages for at least 1 h before starting a new trial.

### Open field test

The open field test was carried out as described previously [18]. Briefly, the mouse was placed in the center of an open-field chamber (40 cm × 40 cm × 40 cm) and was allowed to move freely for 5 min. The movement parameters were monitored and analyzed via a video camera connected to a tracking system (Noldus Ethovision). The ratios of distance, duration, and velocity in the center were measured.

### Real-time PCR

Total RNA was isolated and transcribed to cDNA using a RevertAid™ First-Strand cDNA Synthesis Kit (Fermentas, Glen Burnie, MD, USA), according to the manufacturer’s protocol. Quantitative real-time PCR (qPCR) was performed on an ABI 7500 sequence detector (Applied Biosystems) using SYBR Green I and gene-specific primers (Table 2). Analysis by qPCR included the following steps: a hold step at 50 °C for 2 min to activate uracil-DNA glycosylase with a second hold step at 95 °C for 10 min, followed by 40 cycles at 95 °C for 15 s followed by 60 °C for 1 min. Subsequently, melt analysis was performed by increasing the temperature from 65 to 95 °C. Target gene expression was normalized to GAPDH using the 2^−∆∆CT^ method.

**Table 2:** Related to materials and methods. Mouse primers for real-time PCR

Gene name	Forward primer	Reverse primer
Mouse *Tnf-α*	CAGGAGGGAGAACAGAAACTCCA	CCTGGTTGGCTGCTTGCTT
Mouse *Il-1β*	TCCAGGATGAGGACATGAGCAC	GAACGTCACACACCAGCAGGTTA
Mouse *iNOS*	TAGGCAGAGATTGGAGGCCTTG	GGGTTGTTGCTGAACTTCCAGTC
Mouse *Cox-2*	CAGGCTGAACTTCGAAACA	GCTCACGAGGCCACTGATACCTA
Mouse *Il-6*	GCCAGAGTCCTTCAGAGAGA	GGTCTTGGTCCTTAGCCACT
Mouse *Gapdh*	TGTGTCCGTCGTGGATCTGA	AGGGGCCATCCACAGTCTTC

### Primary cultures

Neuron cultures were obtained from gestational age 13 days C57BL/6J mice embryos as described previously [19]. Dissociated single-cell suspension obtained by mechanical trituration of ventral mesencephalon pieces was seeded on 96-well plates (1 × 10^4^ cells/well).

Mixed glial cultures were obtained from new-born (P0–P2) C57BL/6J mice as described previously [20]. The cerebral cortex was dissociated into a single-cell suspension by treatment with 0.25% trypsin (Invitrogen Corporation) for 15 min at 37 °C and mechanical disruption. The cells were cultured for 9–10 days in DMEM containing 10% fetal bovine serum. Microglia were separated by shaking (200 rpm, 4 h, 37 °C) and added to day 4 primary neuronal cultures grown on 96-well plates (1 × 10^5^ cells/well). Control neuronal cultures were supplemented with an equal volume of cell-free medium. Twenty-four hours later, the cocultures were treated with 500 μM MPP^+^ directly for 12 h [21, 22].

### Neurite outgrowth assay

Primary neuron cultures were incubated with a primary antibody against microtubule-associated protein 2 (MAP2, 1:1000, Abcam) and Alexa Fluor 568-conjugated secondary antibody. MAP2-positive cells were scanned with a Cellomics Kinetic Scan reader (Thermo Fisher Scientific, Waltham, MA, USA). Extended Neurite Outgrowth software (Thermo Fisher Scientific) was used for image analysis.

### Calcium imaging

Purified cells were loaded with Fluo-4 AM (1.6 μM; Beyotime Institute of Biotechnology, Haimen, China) for 25 min in phosphate-buffered saline (PBS) at 37 °C. Ethylene glycol-bis (β-aminoethylether)-*N*,*N*,*N*′,*N*′-tetraacetic acid (2 μM; Tocris Bioscience, Bristol, UK) was used to chelate the calcium in the DMEM. The sarcoplasmic/ER Ca^2+^ ATPase pump blocker thapsigargin (Tg, 2 μM; Tocris Bioscience) was used to induce intracellular calcium release. CaCl_2_ (2 mM) was then added to the DMEM to induce Ca^2+^ influx. Fluorescence signals were recorded and analyzed using a FlexStation 3 multi-mode microplate reader (Molecular Devices, Sunnyvale, CA, USA).

### Measurement of reactive oxygen species

MPP^+^ was added to primary cultured microglia. The microglia were then loaded with 5-(and-6)-chloromethyl-2′,7′-dichlorodihydrofluorescein diacetate (CM-H_2_DCFDA, 30 μM; Invitrogen Corporation) to measure the generation of reactive oxygen species (ROS).

### Immunostaining

Frozen brain tissues were blocked with 10% goat serum in 0.01 M PBS for 1 h and then incubated overnight at 4 °C with primary antibodies (Abs). Brain sections (20 μm) were incubated with the following primary Abs: mouse anti-KCa3.1 (1:100; Alomone Labs, Ltd., Jerusalem, Israel) and rabbit anti-Iba1 (1:500; Wako Pure Chemical Industries, Ltd., Osaka, Japan). The brain sections were then washed with 0.01 M PBS and incubated with the respective Alexa Fluor® 488- or 568-conjugated secondary Abs (1:500; Invitrogen Corporation). Fluorescent images were acquired using a TCS SP8 confocal laser scanning microscope (Leica Microsystems, Wetzlar, Germany). For imaging acquisition, a prescan of all samples was performed to ensure confocal settings below saturation. For each experiment, all images were obtained using the same confocal settings. Six slices at 120 μm intervals from each brain were used to examine Iba1-positive cells. Three microscopic fields (0.01 mm^2^) were randomly selected in each slice with the same reference position for quantification. The Iba1-positive cell number was counted in a blinded manner, and the area was measured by Leica LAS AF Lite software (Leica, Germany).

### Tyrosine hydroxylase immunohistochemistry

Coronal sections through the SNpc were processed for tyrosine hydroxylase (TH) immunohistochemistry [23]. A series of brain coronal sections (20 μm thickness) of the SNpc (− 2.80 to − 3.80 mm to Bregma) were obtained, and then six slices at 120 μm intervals from each brain were used. Briefly, the 20-μm SNpc sections were incubated with rabbit anti-TH (1:500, Millipore, Billerica, MA, USA) at 4 °C overnight. On the second day, sections were treated with biotinylated anti-rabbit IgG and then processed with avidin-biotin peroxidase complex. The peroxidase reaction was visualized by 0.05% DAB + 0.03% H_2_O_2_. A Nikon TE300 inverted microscope was used to measure TH-positive areas in SNpc. Briefly, the number of TH-positive neurons in each section (both the left and right side) was counted by NIH ImageJ [24], and then averaged over the total number of sections per animal [25].

### Western blotting

Total brain tissues were sonicated using a LabSonic homogenizer (B. Braun Biotech Inc., Allentown, PA, USA), and the protein concentration in the brain samples was then quantified using a bicinchoninic acid assay kit (Pierce, CA, USA). Samples were then analyzed by 10% (*w/v*) sodium dodecyl sulfate-polyacrylamide gel electrophoresis and transferred to polyvinylidene difluoride membranes. The membranes were blocked for 1 h with 5% milk containing 0.05% Tween in PBS. The blots were then incubated overnight at 4 °C with the following primary antibodies: β-actin (1:3000; Sigma-Aldrich), rabbit anti-mTOR, rabbit anti-phospho-mTOR (Ser2448), rabbit anti-GRP78, mouse anti-CHOP, rabbit anti-phospho-Akt (Ser473), rabbit anti-phospho-Akt (Thr308), rabbit anti-Akt, rabbit anti-phospho-4E-BP1, rabbit anti-phospho-p70 S6 (1:1000; Cell Signaling Technology, Danvers, MA, USA), and mouse anti-KCa3.1 (1:100; Alomone Labs, Ltd., Jerusalem, Israel). Membranes were then probed with secondary horseradish peroxidase-conjugated antibodies (1:3000; Amersham Biosciences, Little Chalfont, UK) for 1 h at room temperature. The blots were then visualized using chemiluminescent peroxidase substrate (ECL prime; GE Healthcare). Immunoreactivity for each protein band intensity was quantified using NIH ImageJ software [24] and normalized to β-actin as a loading control.

### Statistical analysis

All data are presented as mean ± standard error of the mean. Statistical analyses were performed using Prism software (GraphPad Software, Inc., La Jolla, CA, USA). Data were tested for Gaussian distribution with the Kolmogorov-Smirnov normality test and then analyzed by one-way analysis of variance (ANOVA) and Dunnett’s post hoc test. For the rotarod test comparisons, two-way ANOVA with a Bonferroni post-test was used. Data were analyzed with an unpaired, two-tailed Student’s *t* test when comparing between two groups; if the variable failed the normality test, the non-parametric Mann-Whitney *U* test was applied. Statistical significance was set at *p* < 0.05.

## Results

### Upregulation of KCa3.1 expression in the brains of PD mouse model

**Fig. 1:**
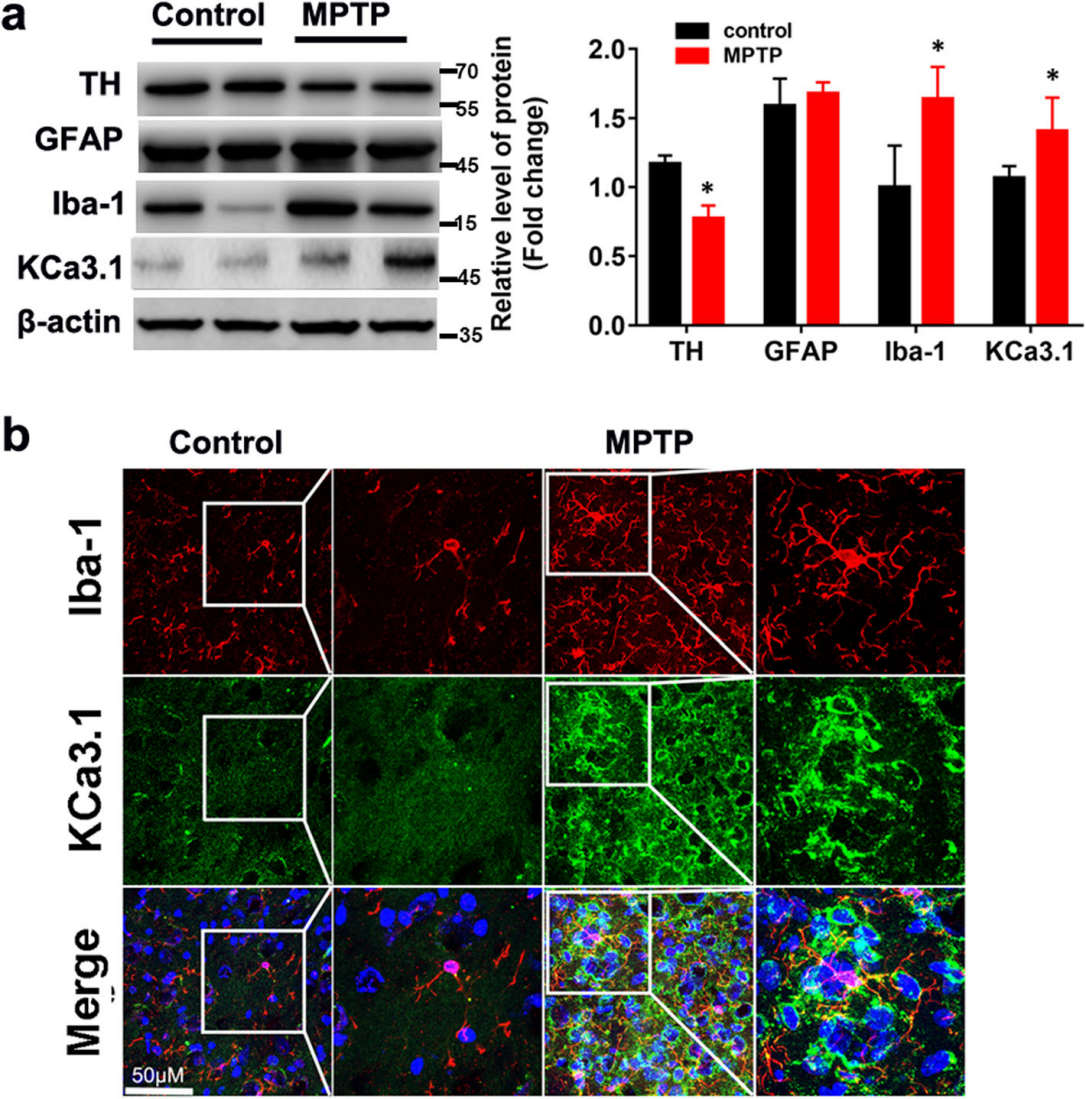
Upregulation of KCa3.1 channels and Iba1 in the brains of PD mouse model. **a** Western blot analysis of SNpc lysates from control and MPTP-induced PD mouse model analyzed by antibodies to TH, GFAP, Iba1, and KCa3.1. Data represent the mean ± SEM (*n* = 3). Western blot was repeated three times and showed similar results; *p < 0.05, unpaired, two-tailed Student’s *t* test compared with control. **b** Double immunofluorescence analysis of KCa3.1 (green) levels in microglia (Iba1, red) of control and MPTP group mouse SNpc. DAPI (blue) was used to label nuclei. Scale bar 50 μm

The mouse model for PD was induced by administration of MPTP as described previously [16]. The expressions of both KCa3.1 and active microglia marker ionized calcium-binding adapter molecule 1 (Iba1) were significantly increased in the SNpc of MPTP-induced PD mice as detected by western blotting (Fig. 1a). While the expression of TH^+^ neurons in SNpc of MPTP group mice was lower than that of control group mice (Fig. 1a). There was no obvious difference in the astrocytic marker glial fibrillary acidic protein (GFAP) expression between the MPTP-induced and control group. Co-immunostaining of KCa3.1 specific for microglia was performed on brain sections of the control and MPTP-induced group mice. In control mice, little level of expression of KCa3.1 was detected in Iba1^+^ microglia (Fig. 1b). However, in MPTP group mice, we detected a clear co-localization between KCa3.1 and Iba1^+^ active microglia in the SNpc regions (Fig. 1b).

### Genetic KCa3.1 deletion and pharmacological blockade reduced MPTP-induced loss of DA neurons

**Fig. 2:**
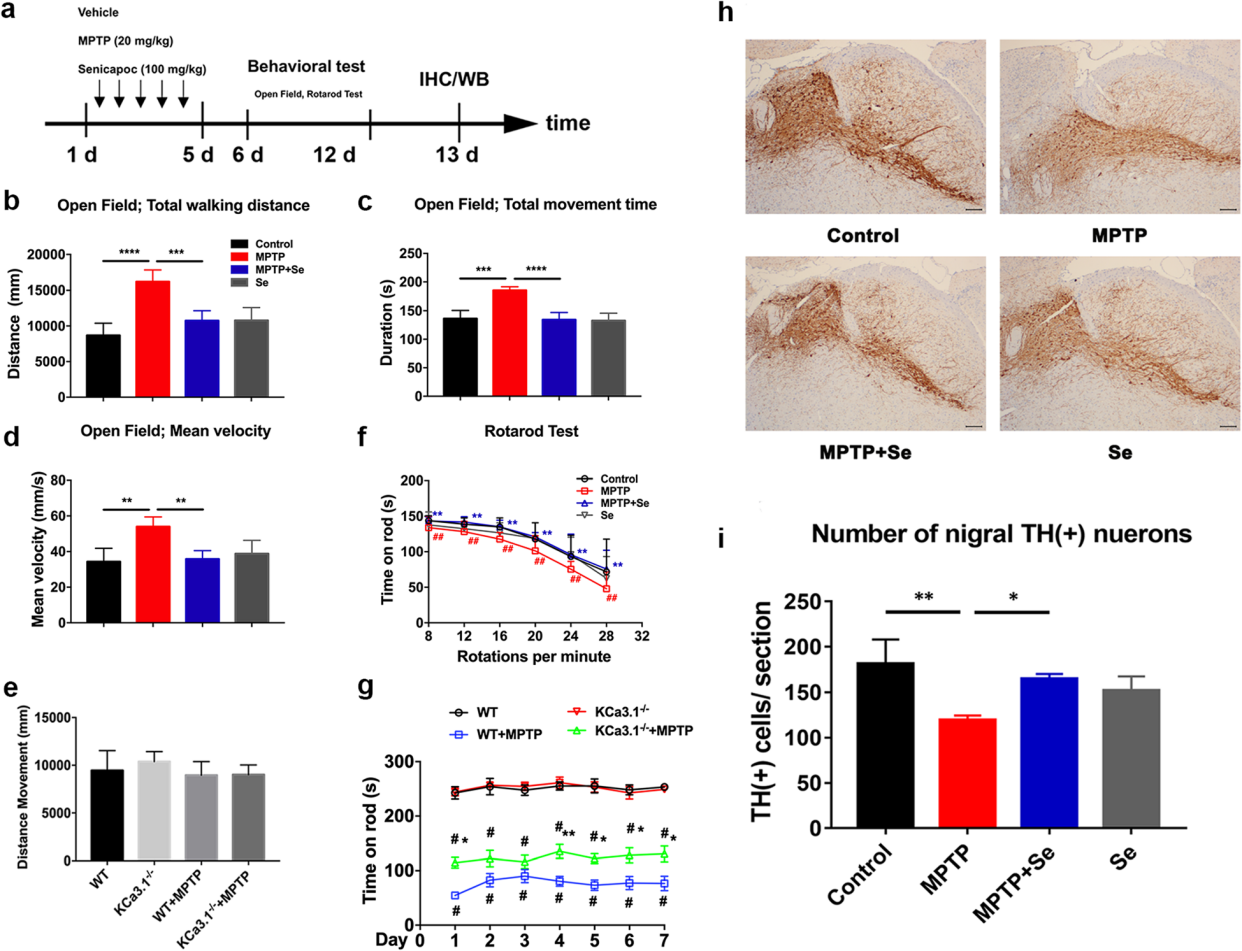
Genetic KCa3.1 deletion and pharmacological blockade with senicapoc attenuate MPTP-induced loss of DA neurons. **a**–**g** WT or KCa3.1^−/−^ mice received sequential intraperitoneal injections of MPTP (20 mg/kg) with or without senicapoc (100 mg/kg, once daily, p.o.) treatment for 5 days as described in the “Material and methods” section. Open field test (**b**–**e**) and the rotarod test (**f**, **g**) for bradykinesia were performed. Behavioral tests for MPTP-induced bradykinesia were conducted on the indicated days. Data are presented as mean ± SEM (*n* = 10–15). **b**–**e** **p < 0.01, ***p < 0.001, ****p < 0.0001, one-way ANOVA followed by the Dunnett’s multiple comparison test. **f**, **g** ^#^*p* < 0.05, ^##^*p* < 0.01 compared to respective control. *p < 0.05, **p < 0.01 compared to the MPTP group. Two-way ANOVA followed by the Bonferroni multiple comparison test. **h** The representative slices of the control and the treatment (MPTP, MPTP+Se, Se) mice. Scale bar 100 μm. **i** Quantitative analysis of TH-positive cells in SNpc. Data are presented as mean ± SEM, *n* = 5, *p < 0.05, **p < 0.01, one-way ANOVA followed by the Dunnett’s multiple comparison test

Microgliosis is known to be associated with neurotoxicity and is involved in the pathological process of PD. Therefore, we employed KCa3.1-specific blocker senicapoc (Fig. 2a–d, f) and KCa3.1^−/−^ mutant mice (Fig. 2e, g) to explore the function of KCa3.1 in the MPTP-induced PD mouse model. The open field test (Fig. 2b–e) and rotarod test (Fig. 2f, g) were conducted daily after MPTP injection. As shown in Fig. 2, treatment with MPTP resulted in a longer distance movement (Fig. 2b), duration (Fig. 2c), and higher mean velocity (Fig. 2d) in the open field test and a shorter time in the rotarod test (Fig. 2f, g). These behavioral impairments were attenuated in MPTP+senicapoc group mice (Fig. 2b–d, f) and KCa3.1^−/−^+MPTP group mice (Fig. 2g). In agreement with this observation, the number of TH^+^ neurons in SNpc of MPTP+senicapoc group mice was higher than that of the MPTP group mice (Fig. 2h, i).

### Genetic KCa3.1 deletion and pharmacological blockade reduced MPTP-induced microglial activation and neuroinflammation

**Fig. 3:**
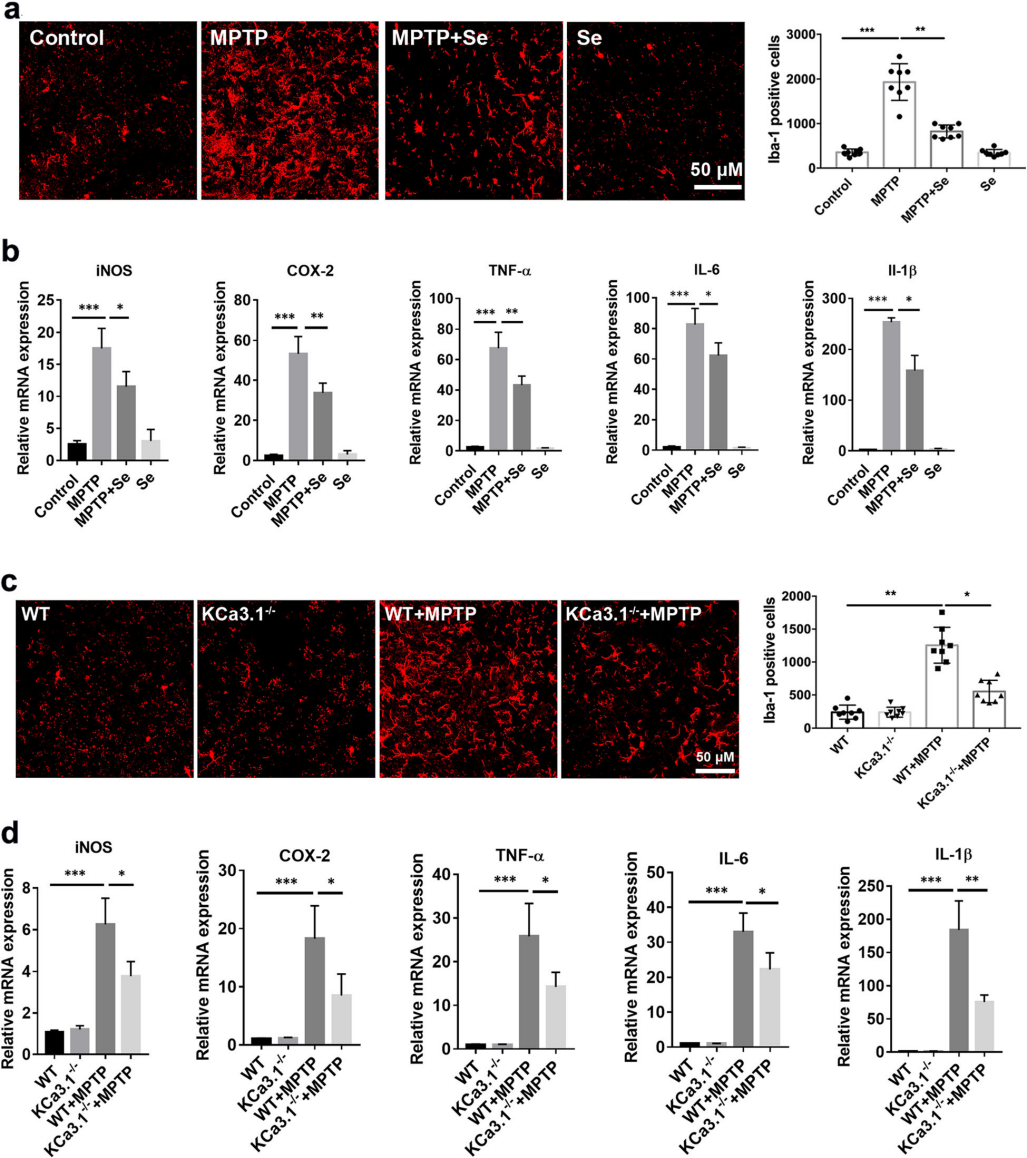
Genetic KCa3.1 deletion and pharmacological blockade with senicapoc attenuate MPTP-induced microgliosis. **a**, **c** Immunostaining for Iba1 in SNpc. Bar 50 μM. Quantitative analysis of Iba1^+^ cells in SNpc. Data are presented as mean ± SEM (*n* = 5–8). *p < 0.05, **p < 0.01, ***p < 0.001. One-way ANOVA followed by the Dunnett’s multiple comparison test. **b**, **d** Expression of iNOS, COX-2, TNF-a, IL-6, and IL-1β in SNpc was measured by qPCR. Data are presented as mean ± SEM (*n* = 5). *p < 0.05, **p < 0.01, ***p < 0.001. One-way ANOVA followed by the Dunnett’s multiple comparison test

The neuroinflammation and neurotoxicity associated with microgliosis are involved in the pathogenesis of neurodegeneration. We investigated whether KCa3.1 gene deletion or pharmacological blockade attenuates microglial activation in MPTP-induced PD mice by measuring the microglia marker Iba1 expression. Our data show that the microglia marker Iba1^+^ cell was increased in the brains of WT+MPTP mice, as compared with that of the control (Fig. 3a) or WT mice (Fig. 3c). Protein level of Iba1 in SNpc was decreased in the MPTP+senicapoc group mice compared to the MPTP group mice (Fig. 3a). Iba1 protein expression in SNpc was also decreased in KCa3.1^−/−^+MPTP group mice as compared with WT+MPTP group mice (Fig. 3c).

Pharmacological blockade of KCa3.1 with senicapoc attenuated the upregulation of iNOS, COX-2, TNF-α, IL-6, and IL-1β in SNpc compared with the MPTP group mice (Fig. 3b). KCa3.1 deficiency also resulted in the downregulation of inflammatory mediators in SNpc following MPTP injection (Fig. 3d). This suggested that KCa3.1-regulated microglia activation may be responsible for the MPTP-induced enhancement of DA neuron death.

### Genetic KCa3.1 deletion and pharmacological blockade reduced MPTP-induced ER stress

**Fig. 4:**
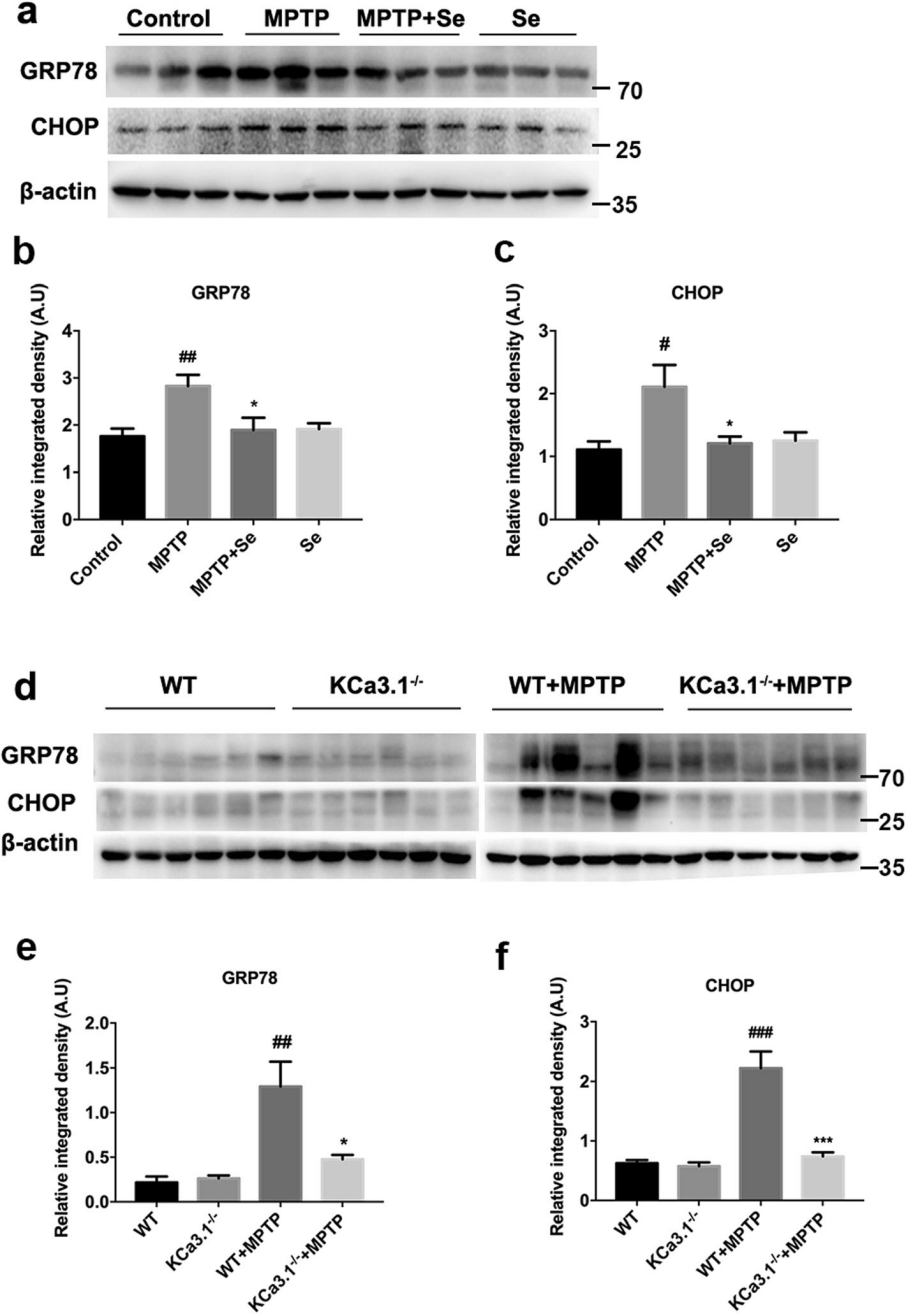
Genetic KCa3.1 deletion and pharmacological blockade with senicapoc attenuated MPTP-induced ER stress. **a**, **d** Western blot analysis of GRP78 and CHOP protein levels in SNpc. **b**, **c**, **e**, **f** Data are presented as the mean ± SEM (*n* = 5–6). Western blot was repeated three times and showed similar results. ^#^*p* < 0.05, ^##^*p* < 0.01, ^###^*p* < 0.001 vs. control or WT group mice; *p < 0.05, ***p < 0.001 vs. MPTP or WT+MPTP group mice. One-way ANOVA followed by Dunnett’s multiple comparison test. WT, wild-type

Western blot analysis was conducted to identify the changes of glucose-regulated protein 78 (GRP78) and CCAAT/enhancer-binding protein homologous protein (CHOP) expression patterns in SNpc of MPTP or WT+MPTP mice, as compared with control or WT mice. GRP78 and CHOP levels were significantly increased in MPTP (Fig. 4a) or WT+MPTP mice (Fig. 4d), as compared to control or WT mice, while the GRP78 (Fig. 4a, b) and CHOP (Fig. 4a, c) expressions were significantly decreased in the MPTP+Se group mice, as compared to the MPTP group mice. Similar to previous results in pharmacological blockade of KCa3.1 with senicapoc, gene deletion of KCa3.1 attenuated MPTP-induced upregulation of GRP78 (Fig. 4d, e) and CHOP (Fig. 4d, f) in SNpc compared with the WT+MPTP group mice.

### Genetic KCa3.1 deletion and pharmacological blockade activated the AKT/mTOR pathway

The foregoing data suggest that genetic deletion or pharmacological blockade of KCa3.1 prevented activation of the unfolded protein response (UPR) and attenuated ER stress in an in vivo MPTP-induced PD model. However, the signaling intermediates linking KCa3.1 and ER stress in PD remain unknown.

**Fig. 5:**
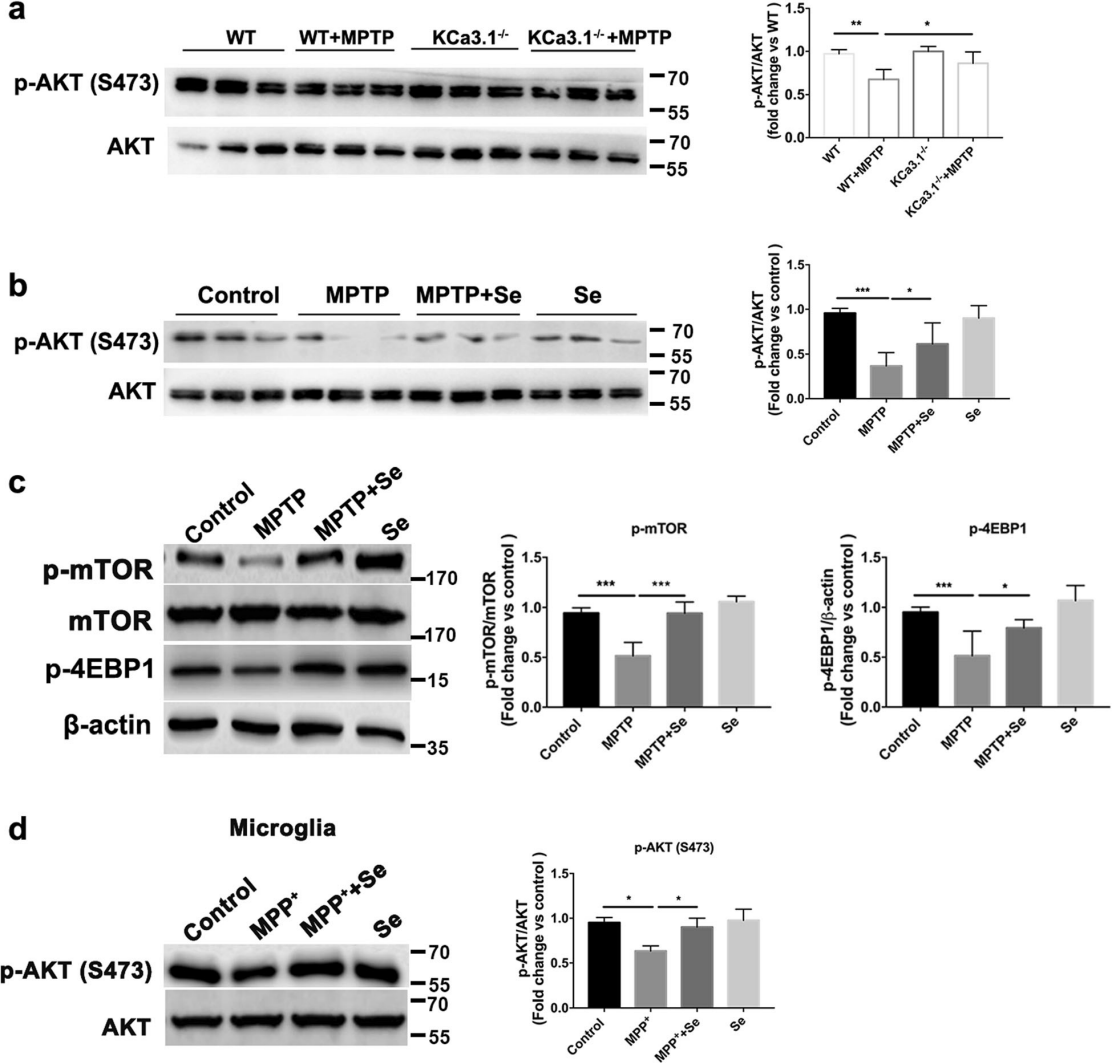
AKT modulation is crucial for KCa3.1-mediated ER stress in microglia. **a**, **b** Representative blots of p-AKT and total AKT in SNpc from **a** WT, WT+MPTP, KCa3.1^−/−^, KCa3.1^−/−^+MPTP group mice and from **b** control, MPTP, MPTP+Se, Se group mice. Data are presented as the mean ± SEM (*n* = 3–5). Western blot was repeated three times and showed similar results. The OD value of p-AKT was normalized to that of AKT. *p < 0.05, **p < 0.01, ***p < 0.001, one-way ANOVA followed by Dunnett’s multiple comparison test. **c** Representative blots of p-mTOR, p-4EBP1, and total mTOR in SNpc from control, MPTP, MPTP+Se, Se group mice. Data are presented as the mean ± SEM (*n* = 3–5). Western blot was repeated three times and showed similar results. *p < 0.05, ***p < 0.001, one-way ANOVA followed by Dunnett’s multiple comparison test. **d** Representative images of p-AKT (S473) and total AKT in microglia responses to 500 μM MPP^+^ for 12 h with or without 1 μM senicapoc. Mean values of p-AKT relative to AKT. Data are presented as the mean ± SEM (*n* = 3). Western blot was repeated 3 times and showed similar results. *p < 0.05, one-way ANOVA followed by the Dunnett’s multiple comparison test. WT, wild-type

We then examined whether gene deletion or pharmacological blockade of KCa3.1 would activate the AKT/mTOR pathway in an in vivo MPTP-induced PD model. p-AKT (Ser473) levels were decreased in SNpc of WT+MPTP (Fig. 5a) or MPTP mice (Fig. 5b). KCa3.1 gene deletion in MPTP mice (KCa3.1^−/−^+MPTP mice) (Fig. 5a) and senicapoc treatment (MPTP+senicapoc mice) (Fig. 5b) attenuated MPTP-induced suppression of p-AKT (Ser473). The phosphorylation of mTOR (p-mTOR) was decreased in SNpc of MPTP mice (Fig. 5c). The suppression of p-mTOR, in turn, inhibited its downstream proteins, including phosphorylated 4EBP1 (p-4EBP1), while blockade KCa3.1 with senicapoc in MPTP mice attenuated the suppression of p-mTOR.

Consistent with the in vivo results, MPP^+^ treatment for 24 h significantly decreased p-AKT (Ser473) level without inducing changes in total AKT in primary cultured microglia, while pharmacological blockade of KCa3.1 with senicapoc inhibited the decrease in p-AKT after MPP^+^ treatment (Fig. 5d).

### Genetic KCa3.1 deletion and pharmacological blockade decreased SOCE-induced Ca^2+^ overload and attenuated ER stress in primary microglia

**Fig. 6:**
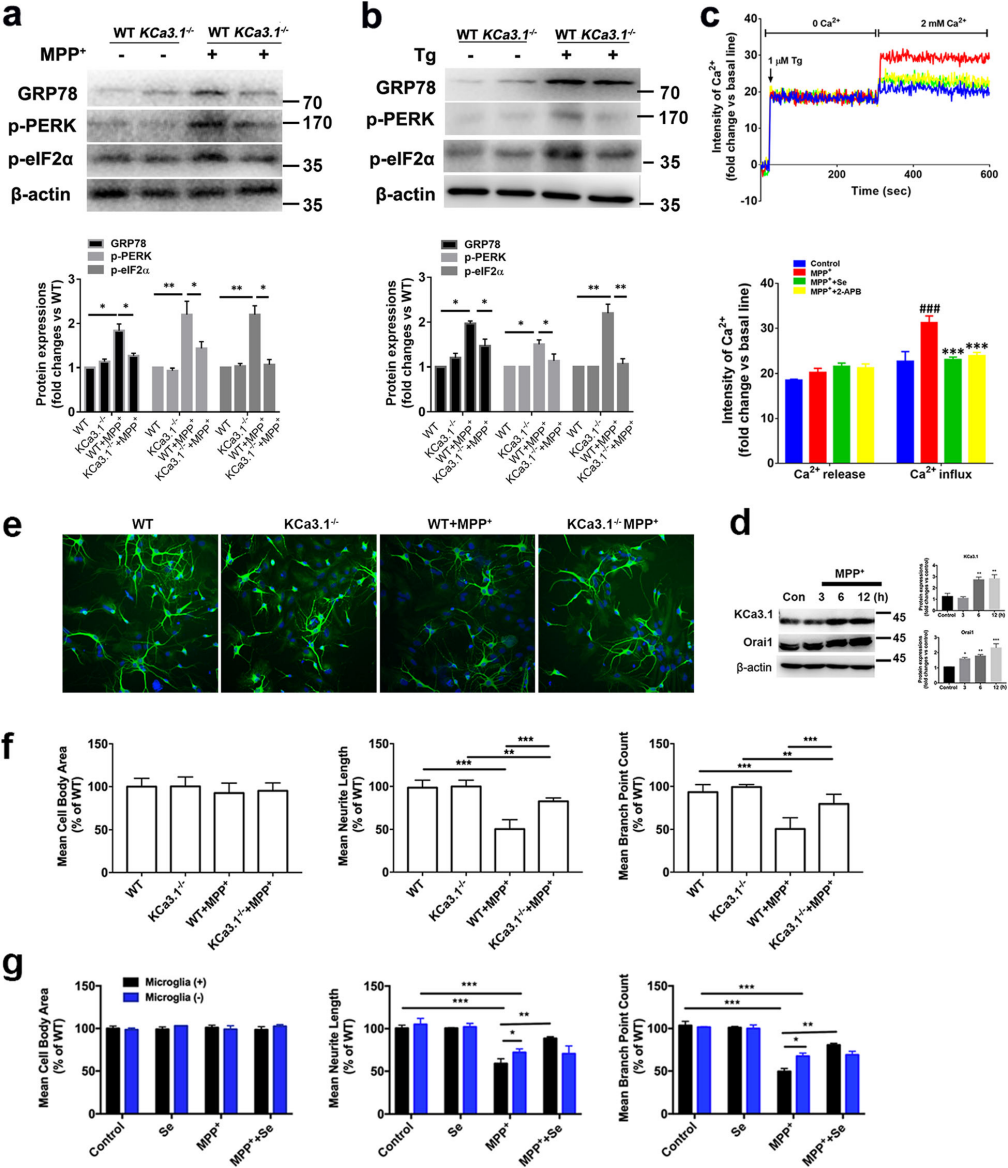
KCa3.1 involved in microglia SOCE and ER stress. **a**, **b** Representative images of GRP78, p-PERK, and p-eIF2α in KCa3.1^−/−^ microglia, responses to 500 μM MPP^+^ (**a**) or 1 μM Tg (**b**) vs. WT cells. Mean values of GRP78, p-PERK, and p-eIF2α relative to β-actin. Data are presented as the mean ± SEM (*n* = 3). Western blot was repeated three times and showed similar results. *p < 0.05, **p < 0.01, unpaired, two-tailed Student’s *t* test. **c** Primary cultured microglia were treated with 500 μM MPP^+^ for 12 h with or without pretreatment of 1 μM senicapoc or 10 μM 2-APB. Fluorescence intensities of [Ca^2+^]_i_ are shown. Fluorescence intensity was measured in the presence of 1 μM Tg with or without 2 mM Ca^2+^. Data are presented as the mean ± SEM (*n* = 10). ^###^*p* < 0.001 vs. control, ***p < 0.001 vs. MPP^+^-treated cells. One-way ANOVA followed by Dunnett’s multiple comparison test. **d** Western blot analysis of KCa3.1 and Orai1 expression after 500 μM MPP^+^-treatment for 3, 6, 12 h. Data represent the mean ± SEM of KCa3.1 and Orai1 density normalized to β-actin values for *n* = 3 cultures. *p < 0.05, **p < 0.01, ***p < 0.001, one-way ANOVA followed by Dunnett’s multiple comparison test compared with control. **e**–**g** Levels of the dendritic marker MAP2 were compared between neurons cocultured with KCa3.1^−/−^ (**e**, **f**) or control microglia (**g**). **e** Neuron dendrites were immunostained with MAP2, and nuclei were stained with DAPI (blue). **e**, **f** Neuron cocultured with WT or KCa3.1^−/−^ microglia treated with 500 μM MPP^+^ for 12 h. **g** Neuron cocultured with/without microglia treated with 500 μM MPP^+^ for 12 h with pretreatment of 1 μM senicapoc for 30 min. Cell body area, neurite length, and branch point counts were analyzed by extended neurite outgrowth bioapplication software. Data represent mean ± SEM (*n* = 3). *p < 0.05, **p < 0.01, ***p < 0.001. One-way ANOVA followed by Dunnett’s multiple comparison test. Tg, thapsigargin; Se, senicapoc

Gene deletion and pharmacological blockade of KCa3.1 with senicapoc were then conducted to confirm the critical role of KCa3.1 in Ca^2+^ homeostasis and ER stress. KCa3.1 gene deletion decreased 500 μM MPP^+^ or 1 μM Tg-induced upregulation of GRP78, as compared to MPP^+^ (Fig. 6a) or Tg-treated WT cells (Fig. 6b). Phosphorylation of PKR-like ER kinase (p-PERK), a critical transducer of the UPR, was increased after stimulation with 500 μM MPP^+^ or 1 μM Tg, but decreased in KCa3.1^−/−^ microglia stimulated with MPP^+^ (Fig. 6a) or Tg (Fig. 6b). Similarly, eukaryotic initiation factor 2α (eIF2α) was also increased in MPP^+^ or Tg-treated WT microglia, but was restored to normal in MPP^+^ or Tg-treated KCa3.1^−/−^ cells (Fig. 6a, b).

Primary cultured microglia were treated with 500 μM MPP^+^ for 12 h with or without pretreatment of 1 μM senicapoc or 10 μM calcium release-activated calcium channel protein 1 (Orai1) inhibitor 2-APB (Fig. 6c). The results showed that senicapoc or 2-APB attenuated MPP^+^-induced SOCE-induced Ca^2+^ overload compared to MPP^+^-treated cells (Fig. 6c). To investigate KCa3.1 activity and the involvement of Orai1 and SOCE channels in neuroinflammation, we stimulated microglia with 500 μM MPP^+^ for 3, 6, or 12 h. As shown in Fig. 6d, MPP^+^ induced a time-dependent upregulation of both KCa3.1 and Orail protein expression (Fig. 6d).

### Genetic KCa3.1 deletion decreased microgliosis-induced neurotoxicity

WT and KCa3.1^−/−^ microglia were used to test whether KCa3.1 was involved in the microgliosis-induced neurotoxicity. More dendritic loss was observed in the neuron and WT microglia coculture system with 500 μM MPP^+^ treatment 12 h (WT+MPP^+^) than in the neuron and KCa3.1^−/−^ microglia coculture system (KCa3.1^−/−^+MPP^+^), as shown by the dendritic marker microtubule-associated protein 2 (MAP2) immunofluorescent staining (Fig. 6e, f). WT+MPP^+^ decreased total neurite length and branch point count (Fig. 6f). KCa3.1^−/−^+MPP^+^ attenuated the effect of WT+MPP^+^ by increasing total neurite length and the branch point count. In microglia-free, neuron-enriched cultures, pre-treatment with senicapoc (30 min before 500 μM MPP^+^ treatment) had no protective effect (Fig. 6g). By contrast, in cocultures of neurons and microglia, senicapoc blocked 500 μM MPP^+^-induced decrease of total neurite length and branch point count (Fig. 6g). The molecular probe CM-H2DCFDA was used to determine the ROS production in MPP^+^-induced microgliosis (Additional file 1: Figure S1).

## Discussion

In the present study, we have demonstrated that gene deletion or pharmacological blockade of KCa3.1 with senicapoc results in improved locomotor ability and the TH-positive neuron number and attenuates the microglial activation and neuroinflammation in the SNpc of MPTP-induced PD mouse model. The involvement of KCa3.1/Orai1 in LPS-mediated Ca^2+^ overload probably contributed to the increased inflammatory response of reactive microgliosis via the AKT/mTOR pathways.

Activated microgliosis was shown in the substantia nigra, striatum, hippocampus, and cortex of autopsy brains from both human brains and animal models of PD [26]. Reports using rodent models of disease also demonstrate upregulation of microgliosis correlating with loss of dopaminergic neuron were reported in both the 6-hydroxydopamine toxin injection rat model and LPS- or MPTP-injected rodent model of PD [27]. Collectively, histologic data of human brains and rodent studies support that microglia activation and the associated microgliosis-induced neuroinflammatory are part of the PD process.

It was reported that MPP^+^ treatment significantly increased the phosphorylation of PI3K/AKT/GSK-3β, reactive oxygen species generation, and p65 activation in BV-2 cells and primary microglia [28]. Microglial activation involves diverse Ca^2+^ signaling-dependent functions that can orchestrate the inflammatory response to CNS injury, and numerous receptors can evoke elevation of intracellular Ca^2+^ [29]. K^+^ efflux resulting from KCa3.1 channel activation leads to membrane hyperpolarization, which in turn facilitates Ca^2+^ influx [30]. Selective activation of KCa3.1 and Ca^2+^ channel Orai1 promotes Ca^2+^ signaling and activation of microglia [31]. In our study, 500 μM MPP^+^ induced a time-dependent upregulation of both KCa3.1 and Orai1 (Fig. 6d). Blockade of KCa3.1 or Orai1 with senicapoc or 2-APB attenuated MPP^+^-stimulated SOCE-induced Ca^2+^ overload compared to MPP^+^-treated cells (Fig. 6c).

Studies of isolated mitochondria have shown that MPP^+^ concentrations of about 10–20 mM need to be reached in the mitochondrial matrix for inhibition of complex I [32–34]. After MPTP treatment, the brain homogenates MPP^+^ concentration can reach more than 10 μM in vivo [33]. The concentration of MPP^+^ required to cause toxicity may vary in different cell types. Catecholaminergic neurons express two transporters, DA transporters (DATs) and vesicular monoamine transporter-2 (VMAT-2), which accumulate MPP^+^ in a positive, unidirectional manner [35]. Finally, the concentration of MPP^+^ in mitochondria is nearly 2000 times higher than brain homogenates, resulting in retaining the toxicant for a sufficient period of time and at high enough concentrations to cause neuron damage. Meanwhile, the MPP^+^ in mitochondria of microglia can reach millimolar level by the ways of passive bidirectional transporter OCT-3 and electrochemical accumulation [33, 36]. However, MPP^+^ is rapidly cleared (1–2 h) due to the bidirectional carrier OCT-3 mediated cytosolic MPP^+^ efflux [37]. When extracellular MPP^+^ is present in the low micromolar range, OCT-3 is inadequate to enable quantitative complex I inhibition in microglia.

SOCE channels are complexes composed of the ER calcium sensor stromal interaction molecule 1 and the pore-forming protein Orai1. SOCE channels can be activated by Ca^2+^ store depletion in the ER, which regulates [Ca^2+^]_i_ homeostasis and protein folding [38]. SOCE plays an important role in the process of non-excitable cell activation such as microglia through triggering Ca^2+^ influx [39]. Protein folding disruption of the ER triggers the UPR via three ER pathways: PKR-like ER kinase (PERK), inositol-requiring enzyme 1 (IRE1), and activating transcription factor 6 (ATF6) [40, 41]. During ER stress, the GRP78 dissociates from PERK, IRE1, and ATF6, and then initiates proapoptotic signaling through the CHOP activation. In our study, KCa3.1 involves in SOCE-induced Ca^2+^ overload (Fig. 6c) and ER stress (Fig. 4) via AKT signaling pathway during microglia activation (Fig. 5). Gene deletion or pharmacological blockade of KCa3.1 restored AKT/mTOR signaling both in vivo and in vitro (Fig. 5)*.*

Senicapoc was previously advanced to a phase 3 clinical trial for sickle cell anemia and was found to be safe and well-tolerated [42–44]. Senicapoc inhibits KCa3.1 channels in human erythrocytes with the IC_50_ of 11 nM. However, it failed to achieve its primary clinical end-point in phase 3, which was reduction in the rate of vaso-occlusive pain crisis [45]. Senicapoc is now deposited in the National Institutes of Health National Center for Advancing Translational Research library (PF-05416266), which makes it available for investigator-initiated clinical trials. Senicapoc exhibited excellent brain penetrance (C_brain_/C_plasma_~5), and oral availability makes it suitable for the potential treatment of neurodegenerative disease [46, 47]. It reduced neuroinflammation, enhanced hippocampal neuronal plasticity, and decreased cerebral amyloid load in AD mouse model (50 mg/kg, p.o.) [46]. Inhibition of KCa3.1 by senicapoc reversed tactile allodynia in rats with peripheral nerve injury (100 mg/kg, p.o.) [48].

The expression of KCa3.1 channels has been demonstrated in primary microglia, but not in the unperturbed CNS tissue given the specificity issues with available KCa3.1 antibodies [49]. There was evidence that microglia express KCa3.1 under ischemic pathological conditions and AD [46, 50]. We detected a clear co-localization between KCa3.1 and microglia in the SNpc regions of MPTP group mice (Fig. 1b). In the present studies, we report that genetic KCa3.1 deletion and senicapoc (100 mg/kg, p.o.) reduced MPTP-induced loss of DA neurons (Fig. 2h), microgliosis (Fig. 3a), and the upregulation of iNOS, COX-2, TNF-α, IL-6, and IL-1β in SNpc compared with the MPTP group mice (Fig. 3b). Considering that primary culture microglia resembles a reactive phenotype in vitro and that intraventricular LPS injection induces upregulation of KCa3.1 in vivo [10], we could speculate that KCa3.1 might be exploited as a therapeutic target in microgliosis diseases [51, 52]. In conclusion, our preclinical data support that senicapoc has the potential to expedite the urgently needed new drug discovery for PD.

## Conclusions

The current study established that pharmacological blockade or gene deficiency of KCa3.1 reduces the MPP^+^-induced pro-inflammatory response in cultured primary microglia. In vivo, KCa3.1 deficiency reduces the locomotor ability deficits, TH-positive neuron loss, microglial activation, and neuroinflammation in the SNpc of MPTP-induced PD mouse model. Together, our data suggest that KCa3.1 might be an effective target in driving a pro-inflammatory microglia phenotype in PD.

## Supplementary information

**Additional file 1: Figure S1.** ROS levels stimulation with MPP^+^ in microglia. Microglia were stimulated with 1, 10, 50, 100, 500, 1000 μM MPP^+^ for 12 h and ROS levels were measured by DCFH-DA. Data represent mean ± SEM (*n* = 6). ^**^p < 0.01, ^****^p < 0.0001 compared with control group.

## Data Availability

Data sharing is not applicable to this article as no datasets were generated or analyzed during the current study.

## Abbreviations

AKT: The protein kinase B
ATF6: Activating transcription factor 6
CHOP: CCAAT/enhancer-binding protein homologous protein
CNS: Central nerve system
DA: Dopaminergic
DAPI: 4′,6-Diamidino-2-phenylindole
DMEM: Dulbecco’s modified Eagle medium
eIF2α: Eukaryotic initiation factor 2α
ER: Endoplasmic reticulum
GFAP: Glial fibrillary acidic protein
GRP78: Glucose-regulated protein 78
Iba1: Ionized calcium-binding adapter molecule 1
IL-1: Interleukin 1
IRE1: Inositol-requiring enzyme 1
KCa3.1: Intermediate-conductance calcium-activated potassium channel
LPS: Lipopolysaccharide
MAP2: Microtubule-associated protein 2
MPP^+^: 1-Methyl-4-phenylpyridinium
MPTP: 1-Methyl-4-phenyl-1,2,3,6-tetrahydropyridine
mTOR: Mammalian target of rapamycin
Orai1: Calcium release-activated calcium channel protein 1
PBS: Phosphate-buffered saline
PERK: PKR-like ER kinase
SNpc: Substantia nigra pars compacta
SOCE: Store-operated Ca^2+^ entry
Tg: Thapsigargin
TH: Tyrosine hydroxylase
TNF: Tumor necrosis factor
UPR: Unfolded protein response
