# Supplementary figures and images for: The potassium channel KCa3.1 represents a valid pharmacological target for microgliosis-induced neuronal impairment in a mouse model of Parkinson’s disease
Source: J Neuroinflammation. 2019 Dec 26;16:273. doi: 10.1186/s12974-019-1682-2 (PMC6931251; doi:10.1186/s12974-019-1682-2)

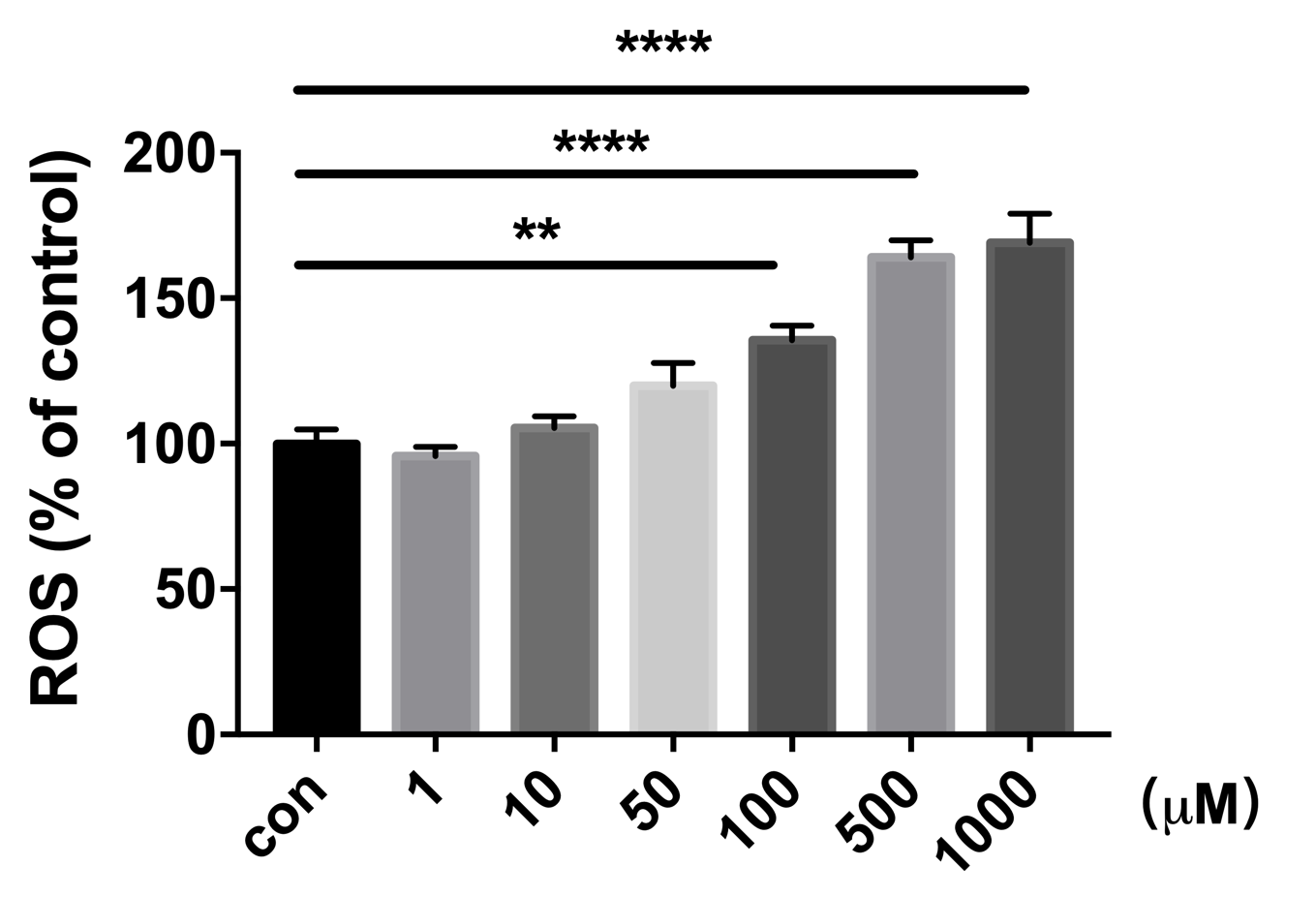

Supplement: Supplementary file 1 — Additional file 1: Figure S1. ROS levels stimulation with MPP+ in microglia. Microglia were stimulated with 1, 10, 50, 100, 500, 1000 μM MPP+ for 12 h and ROS levels were measured by DCFH-DA. Data represent mean ± SEM (n = 6). **p < 0.01, ****p < 0.0001 compared with control group. [file 12974_2019_1682_MOESM1_ESM.tiff]
